# Synergistic Effects of Nisin, Lysozyme, Lactic Acid, and Citricidal^TM^ for Enhancing Pressure-Based Inactivation of *Bacillus amyloliquefaciens*, *Geobacillus stearothermophilus*, and *Bacillus atrophaeus* Endospores

**DOI:** 10.3390/microorganisms9030653

**Published:** 2021-03-21

**Authors:** Sadiye Aras, Niamul Kabir, Sabrina Wadood, Jyothi George, Shahid Chowdhury, Aliyar Cyrus Fouladkhah

**Affiliations:** 1 Public Health Microbiology Laboratory, Tennessee State University, Nashville, TN 37209, USA; saras@tnstate.edu (S.A.); mkabir@tnstate.edu (N.K.); swadood@tnstate.edu (S.W.); jgeorge3@tnstate.edu (J.G.); schowdh1@tnstate.edu (S.C.); 2 Department of Biological Sciences, Tennessee State University, Nashville, TN 37209, USA; 3 Cooperative Extension Program, Tennessee State University, Nashville, TN 37209, USA

**Keywords:** high-pressure processing, bacterial endospores, bioactive compounds

## Abstract

The inactivation of bacterial endospores continues to be the main curtailment for further adoption of high-pressure processing in intrastate, interstate, and global food commerce. The current study investigated the effects of elevated hydrostatic pressure for the inactivation of endospore suspension of three indicator spore-forming bacteria of concern to the food industry. Additionally, the effects of four bacteriocin/bactericidal compounds were studied for augmenting the decontamination efficacy of the treatment. Elevated hydrostatic pressure at 650 MPa and at 50 °C was applied for 0 min (untreated control) and for 3, 7, and 11 min with and without 50K IU of nisin, 224 mg/L lysozyme, 1% lactic acid, and 1% Citricidal^TM^. The results were statistically analyzed using Tukey- and Dunnett’s-adjusted ANOVA. Under the condition of our experiments, we observed that a well-designed pressure treatment synergized with mild heat and bacteriocin/bactericidal compounds could reduce up to >4 logs CFU/mL (i.e., >99.99%) of bacterial endospores. Additions of nisin and lysozyme were able, to a great extent, to augment (*p* < 0.05) the decontamination efficacy of pressure-based treatments against *Bacillus amyloliquefaciens* and *Bacillus atrophaeus*, while exhibiting no added benefit (*p* ≥ 0.05) for reducing endospores of *Geobacillus stearothermophilus*. The addition of lactic acid, however, was efficacious for augmenting the pressure-based reduction of bacterial endospores of the three microorganisms.

## 1. Introduction

According to a Morbidity and Mortality Weekly Report of the U.S. Centers for Disease Control and Prevention, achieving “safer and healthier foods” is one of the top ten main public health achievements of the 20th century [1]. Despite considerable progress, in a typical year in the United States, it is estimated that as many as >3000 individuals lose their lives to foodborne diseases [2]. The global public health burden of foodborne diseases is even grimmer, with some epidemiological studies estimating that in a typical year as many as 600 million individuals contract foodborne diseases, and around 420,000 die due to foodborne infections. The World Health Organization additionally estimates 33 million years of healthy lives are lost every year due to these infections, and around 30% of mortalities occur in children under the age of 5 [3].

The illness, hospitalization, long-term health complications, and death episodes are only one side of the coin. On the other side, foodborne microorganisms are additionally the dominant cause of spoilage and food waste that are not only important public health challenges but also contributors to the production of greenhouse gasses and climate change [4]. The United States Department of Agriculture Economic Research Service estimates around 31% food loss in the United States [5]. The global food waste statistics are similar to those of the United States, where it is estimated that around one third of the world’s food supply goes to waste every year [6]. Challenges associated with foodborne microbial pathogens and spoilage microorganisms are expected to be augmented in the future under the landscape of climate change as increases in environmental temperature have profound effects on the proliferation of various bacteria [4,7,8].

Specifically, concerning the microorganisms used in the current study, it is noteworthy that in addition to causing challenges for spoilage of an array of food products, various species of *Bacillus* are important from a public health perspective as well [9,10]. In the last few decades, the genera and species of these microorganisms have undergone considerable taxonomic changes, and various species of *Bacillus* could lead to morbidity and mortality for healthy people and at-risk individuals including the elderly, the immunocompromised, and children [11]. There are additional concerns that bacteria belonging to the *Bacillus* genus, similar to other bacterial pathogens, are prone to developing antibiotic resistance [12].

While great progress had been made for assuring the safety of food products and reducing food waste [13,14], existing challenges indicate the great need for cutting-edge and innovative technologies for mitigating the risk of foodborne pathogens and spoilage microorganisms. Among various emerging technologies, the application of elevated hydrostatic pressure or high-pressure processing is gaining increasing importance and momentum [15,16]. Although the use of elevated hydrostatic pressure for the inactivation of microorganisms was proposed more than a century ago, in recent years, due to advancements in commercially available pressure-based pasteurizers, this technology is becoming an important part of food commerce [17,18]. High-pressure processing exposes the final packaged products to pressure intensity levels of typically around 650 MPa for durations lasting typically around three minutes [19,20,21]. Recent studies indicate that the use of elevated hydrostatic pressure could not only improve the safety and shelf-stability of a product but could also lead to a product with better organoleptic and nutritional qualities and a more sustainable product compared to traditional heat-treated commodities [22,23]. While this technology has been very efficacious against vegetative cells, one of the main limitations of this technology is the inactivation of bacterial endospores that are inherently resistant to pressure-based treatments. This is currently the main curtailment for these commodities, requiring refrigeration for prolonged shelf-life of pressure-treated products [24,25,26]. 

Considering that high-pressure processing is becoming a leading processing method in manufacturing and considering that inactivation of microbial endospores is the main challenge associated with this emerging technology, our study investigated the effects of this treatment for inactivation of three indicator spore-forming bacteria of food industry concern. Our study additionally investigated effects of four bacteriocin and/or bactericidal compounds to augment the decontamination efficacy of pressure-based treatments.

## 2. Materials and Methods

### 2.1. Bacterial Strains, Propagation, and Endospore Suspension Preparation

The current study utilized three spore-forming bacteria for preparation of the endospore suspensions. *B. amyloliquefaciens* TMW 2.479 (kindly provided by Michael Ganzle from the University of Alberta) had been isolated initially from a ropy bread sample. In the literature, this bacterium has been considered as one of the most capable microorganisms to produce pressure-tolerant spores [27,28,29]. As such, this strain was considered as an indicator microorganism for pressure-based treatments. *B. amyloliquefaciens* was cited as “AMY” in the graphical representations in the current study. *G. stearothermophilus* obtained from American Type Culture Collection (ATCC^®^ 7953^TM^) was the second bacterium used in the study and was cited as “GEO” in the graphical representations in the current study. This bacterium is the causative agent for “flat sour” spoilage in the canning industry and is considered as an indicator microorganism for gas and steam sterilization [30,31]. Lastly, *B. atrophaeus* obtained from American Type Culture Collection (ATCC^®^ 9372^TM^) was used in the current study that is typically considered an indicator microorganism for steam and dry-heat sterilizations [32]. This avirulent bacterium is also considered as a validated surrogate for spore-forming pathogens of public health concern such as *Bacillus anthracis*, the causative agent of anthrax [32]. In the current study’s graphical representations, *B. atrophaeus* is cited as “ATR”.

All three bacteria were stored in glycerol stock at −80 °C [33,34] and were activated by transferring a loopful of the stock, for each microorganism separately, into 10 mL of tryptic soy broth (Difco, Becton Dickinson, Franklin Lakes, NJ, USA) supplemented with 0.6% (i.e., TSB + YE) yeast extract (Difco, Becton Dickinson). The addition of yeast extract had been proposed in the past to minimize the acid stress to the microorganisms [21,35]. The inoculated TSB + YE for *B. atrophaeus* and *B. amyloliquefaciens* were incubated statically at 32 °C for 24 h [36]. For *G. stearothermophilus*, the TSB + YE was incubated at 55 °C for 24 h [30]. After this 24-h aerobic incubation, 0.1 mL from each overnight suspension was aseptically transferred to another 10 mL of sterilized TSB + YE for another 24 h at 32, 32, and 55 °C, for *B. amyloliquefaciens*, *B. atrophaeus*, and *G. stearothermophilus*, respectively [37].

These sub-cultured bacterial suspensions were further used to prepare the endospore suspension based on the method previously validated and utilized for pressure-based pasteurization of bacterial endospores [36,38]. In short, 0.5 mL of each sub-cultured suspension, for each bacterium separately, was aseptically transferred and spread-plated onto the surface of nutrient agar (Difco, Becton Dickinson) supplemented with 0.6% yeast extract and further supplemented with 10 mg/L (i.e., 10 ppm (parts per million)) of MnSO_4_·H_2_O (VWR International, Radnor, PA, USA). The inoculated plates were then incubated again at 32 °C (for *B. amyloliquefaciens* and *B. atrophaeus*) and at 55 °C (for *G. stearothermophilus*) for three days to obtain more than 90% sporulated population on the surface of the medium. This procedure had been validated in the past by observing the below-mentioned harvested endospore suspension under a phase-contrast microscope [36]. After the three-day incubation period, 1.5 mL of sterile deionized water (total dissolved solids <10 mg/L (ppm)) was used for flooding each plate with manual shaking for one minute for harvesting the cells from the medium. This procedure, for each strain separately, was repeated 5 times and the collected suspensions were accumulated into three separate composites for the three strains. The collected bacterial/endospore suspensions were exposed to centrifugal forces (Eppendorf North America, Hauppauge, NY, USA, model 5424, Rotor FA-45-24-11) for 15 min at 5000× rpm (c. 2460× *g* for 88 mm rotor) for harvesting endospores/cells. The supernatant was discarded, and the cells were resuspended in carrot juice (for experiments of trial 1) or sterilized deionized water (for experiments of trial 2), at target inoculation of 5–7 (high inoculation level) and 3–5 (low inoculation level) log CFU/mL, respectively. The resuspended substrate was then, for each bacterium separately, pasteurized at 80 °C for 15 min for eliminating vegetative cells. These endospore suspensions were then kept at 4 °C before the experiments.

### 2.2. High-Pressure Processing and Bacteriocin/Bactericidal Compounds

The current study utilized four bacteriocin and/or bactericidal compounds of nisin, lysozyme, lactic acid, and Citricidal^TM^ to augment the decontamination efficacy of pressure-based treatments at 650 MPa and 50 °C. Currently, food manufacturing facilities rely on treatments of 650 MPa lasting for typically around three minutes. The use of temperature at 50 °C is considered a mild heat treatment, and products treated at this temperature could still be considered to be non-thermally processed [21,39]. The antimicrobials utilized in the current study and their concentrations were selected based on conducted preliminary trials and previously published studies in the public health microbiology program [21,35,40,41]. As an example, in previously conducted trials it has been observed that 5000 IU of nisin was not capable of augmenting the effectiveness of treatments at 650 MPa against bacterial endospores [41]. Thus, this study examined a 10-fold increase in the concentration of this bacteriocin compound (e.g., 50K IU of nisin). All antimicrobials were filter-sterilized by 0.2 µm polyethersulfone membrane (VWR International, Radnor, PA, USA) and were added precisely before treatment and, as explained in Section 2.3, immediately neutralized after each treatment to assure accurate exposure times. Specifically, 50K IU (*w*/*v*) of nisin (MP BioMedicals, LLC, Solon, OH, USA), 224 mg/L (*w*/*v*) of lysozyme (MP BioMedicals, LLC, Solon, OH, USA), 1% (*v*/*v*) lactic acid (Research Products International, Mt. Prospect, IL, USA), and 1% (*v*/*v*) Citricidal^TM^ (NutriBiotic^®^, Lakeport, CA, USA) were used in the current study.

Pressure processing of the samples was conducted inside no-disk pulse tubes (Pressure BioScience Inc., South Easton, MA, USA) with a capacity of 1.5 mL. Samples were treated at pressure intensity of 650 MPa using Hub880 Baracycle unit (Pressure BioScience Inc., South Easton, MA, USA) with automatic monitoring and recording of pressure and temperature values every three seconds using 1.0.8 v. of HUB Explorer PBI software (Pressure BioScience Inc.). The temperature was recorded by a T-type thermocouple (Omega Engineering Inc., Norwalk, CT, USA) inserted inside the chamber wall and connected to the unit software. The temperature was regulated by a stainless-steel water jacket surrounding the pressure chamber that was mechanically connected to a circulating water bath (Model 1160s Refrigerated, VWR International, Radnor, PA, USA). The pressure chamber was purged by a mechanical pump before each analysis for the removal of residual air. Distilled water was used as pressure transmission fluid; thus, the treatments could be considered elevated hydrostatic pressure.

### 2.3. Microbiological Enumeration, Neutralization, and pH Analysis

The current study provides information on two sets of experiments: (i) trials conducted in carrot juice at high inoculation level of the three endospores in the presence of nisin or lysozyme, and (ii) trials conducted in distilled water at a low inoculation level of the three endospores in the presence of lactic acid or Citricidal^TM^. For both experiments, samples were treated using elevated hydrostatic pressure at 650 MPa at 50 °C with and without the above-mentioned antimicrobials for 0 min (untreated control), and 3, 7, and 11 min. To assure precise control of exposure time, each antimicrobial compound was added immediately (c. <3 s) before the pressure treatments, and immediately after analyses, 1 mL of the treated sample was neutralized with sterilized D/E neutralizing broth (Difco, Becton Dickinson). The neutralized suspensions were then immediately placed on ice-water slurry. The detection limit of the current study was 0.60 log CFU/mL. Treated and neutralized samples were used for the measurement of pH using a pH meter (SevenExcellence^TM^ Mettler Toledo AG, Grelfensee, Switzerland) calibrated at pH values of 4, 7, and 10 prior to analyses. Treated and neutralized samples were additionally 10-fold serially diluted in 1X Maximum Recovery Diluent (Difco, Becton Dickinson) to enhance the recovery of injured but viable cells. After homogenizing the diluent with a high-speed (3200× rpm) vortex mixer (Model Vortex-2 Genie, Scientific Industries, Bohemia, NY, USA), each sample was spread-plated onto the surface of tryptic soy agar (Difco, Becton Dickinson) supplemented with yeast extract. Supplementation with yeast extract additionally enhances recovery of pressure/heat/antimicrobial injured but viable cells [21,35]. Spread-plated samples were then aerobically incubated at 32 °C (for *B. amyloliquefaciens* and *B. atrophaeus*) or 55 °C (for *G. stearothermophilus*) for 2 days for enumeration of survivors. The colony-forming units of each sample were then counted manually using a Quebec colony counter, based on U.S. Food and Drug Administration’s Bacteriological Analytical Methods [42].

### 2.4. Design of Trials and Descriptive and Inferential Analytical Methods

Experiments conducted in carrot juice and distilled water were conducted separately, and thus were analyzed and reported independently. Each of these experiments consisted of two biologically independent repetitions, considered as blocking factors in a complete randomized block design. Each block further consisted of two replications and each replication was repeated twice (as instrumental/microbiological replicates). This design is based on sample size and statistical power analyses conducted in public health microbiology program for pressure pasteurization of liquid products [43]. This a priori power analysis exhibited that at least five repetitions are needed to observe the mean difference of 0.15 log CFU/mL as statistically significant differences (power level of 80% and type I error level of 5%, i.e., α = 0.05). Based on the above-mentioned design, each reported value is the mean of eight independent observations (two blocks, two replications per block, and two instrumental/microbiological replicates per replication).

**Figure 1:**
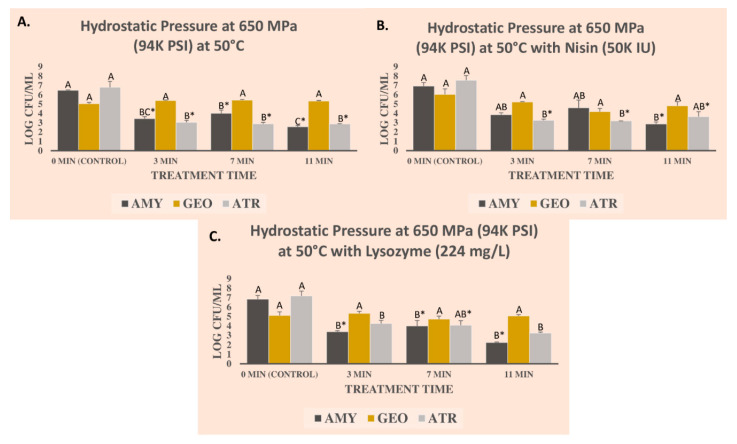
Inactivation of *Bacillus amyloliquefaciens* (AMY), *Geobacillus stearothermophilus* (GEO), and *Bacillus atrophaeus* (ATR) treated with the elevated hydrostatic pressure of 650 MPa at 50 °C in carrot juice. (**A**) Treatments without any added antimicrobial. (**B**) Treated with elevated hydrostatic pressure of 650 MPa at 50 °C with 50K IU (*w*/*v*) nisin. (**C**) Treated with the elevated hydrostatic pressure of 650 MPa at 50 °C with 224 mg/L (*w*/*v*) lysozyme. For each panel of the figure and each bacterium separately, columns followed by different uppercase letters are statistically (*p* < 0.05) different from each other (Tukey-adjusted ANOVA). Columns followed by * sign are statistically (*p* < 0.05) different from the control (Dunnett’s-adjusted ANOVA).

**Figure 2:**
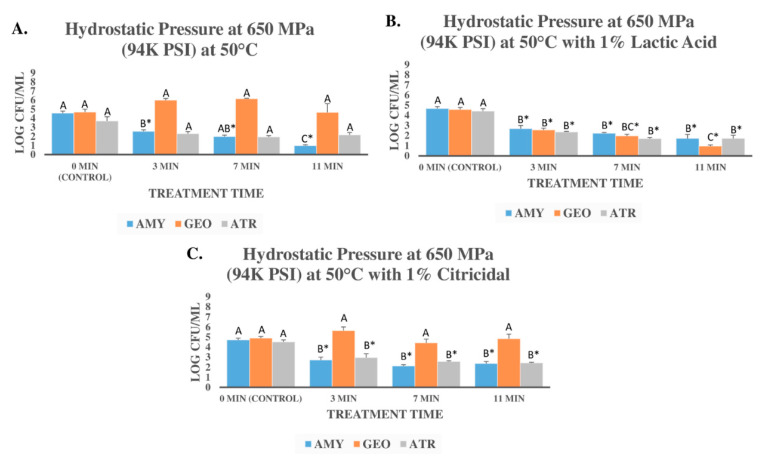
Inactivation of *Bacillus amyloliquefaciens* (AMY), *Geobacillus stearothermophilus* (GEO), and *Bacillus atrophaeus* (ATR) treated with the elevated hydrostatic pressure of 650 MPa at 50 °C in distilled water. (**A**) Treatments without any added antimicrobial. (**B**) Treated with elevated hydrostatic pressure of 650 MPa at 50 °C with 1% (*v*/*v*) lactic acid. (**C**) Treated with the elevated hydrostatic pressure of 650 MPa at 50 °C with 1% (*v*/*v*) Citricidal^TM^. For each panel of the figure and each bacterium separately, columns followed by different uppercase letters are statistically (*p* < 0.05) different from each other (Tukey-adjusted ANOVA). Columns followed by * sign are statistically (*p* < 0.05) different from the control (Dunnett’s-adjusted ANOVA).

Microbial counts obtained from the procedure in Section 2.3 were log-transformed and mean and standard errors were used as descriptive statistics for the preparation of graphical representations. Two types of inferential statistics were additionally calculated for each bacterium separately. After analyses of variance using *Proc glm* of SAS_9.4_ (SAS Inst., Cary, NC, USA), a Tukey-adjusted pair-wise comparison was conducted; thus, in the graphical representations (Figure 1 and Figure 2) for each panel of the figures and each bacterium separately, columns followed by different uppercase letters are statistically different from each other. Additionally, a Dunnett’s-adjusted comparison (comparing each treatment with the control, i.e., 0 min treatments) was conducted. As such, for each panel of the figures and each bacterium separately, columns followed by * sign are statistically different from the control. Similar to the power analyses, Tukey- and Dunnett’s-adjusted ANOVA were conducted at type I error level of 5% (α = 0.05). Additionally, D-values and K_max_ values were calculated using Microsoft Excel and GlnaFiT (Katholieke Universiteit, Leuven, Belgium) v. 1.7 software [44].

## 3. Results and Discussion

The pH values (mean ± standard deviation) of the samples were similar (*p* ≥ 0.05) among the strains and were increased (*p* < 0.05) after neutralization using D/E neutralizing broth. As an example, for the experiment conducted in carrot juice, the pH of *B. amyloliquefaciens*, *G. stearothermophilus*, and *B. atrophaeus* samples were 5.86 ± 0.07, 6.06 ± 0.25, and 5.91 ± 0.06, before neutralization, respectively. These values, for the corresponding order of microorganisms, were 7.33 ± 0.04, 7.35 ± 0.03, and 7.34 ± 0.01 after neutralization (before microbiological analysis), respectively. In the experiment conducted in the distilled water, pH values of the samples after neutralization were also similar (*p* ≥ 0.05) and were 7.44 ± 0.01, 7.42 ± 0.06, and 7.42 ± 0.00 for *B. amyloliquefaciens*, *G. stearothermophilus*, and *B. atrophaeus*, respectively. As further discussed in the Materials and Methods (Section 2.2), the temperature of the treatments was precisely controlled using a stainless-steel water jacket surrounding the pressure treatment chamber that was mechanically connected to a circulated water bath. The temperature was automatically monitored before, during (every three seconds), and after the treatments using a T-type thermocouple inserted inside the chamber wall. The before and after temperatures remained constant (*p* ≥ 0.05) and were not statistically different (*p* ≥ 0.05) from the set point temperature of 50 °C. Control and monitoring of temperature had been one of the main challenges associated with external validity of pressure-based microbiological hurdle validation studies [39]; thus, precise control of temperature assures that observed results are due to effects of pressure and antimicrobial application rather than fluctuations in temperature during processing. Furthermore, neutralizing the samples immediately (<10 s) and placing them on ice-water slurry after treatment ensures that exposure times to heat and antimicrobials are controlled and that there is no residual heat and antimicrobial effect on the surface of the bacteriological medium during enumeration of the microorganisms [19,45].

### 3.1. Pressure-Based Reduction of Three Bacterial Endospores in the Presence of Nisin and Lysozyme

Three bacterial endospores were used in the current study. As previously discussed in Section 2.1., *B. amyloliquefaciens* TMW 2.479 was selected as an indicator for pressure-based treatments. In experiments conducted in carrot juice (Figure 1) endospore counts of the bacterium before treatment with 650 MPa elevated hydrostatic pressure at 50 °C were 6.48 ± 0.06 (mean ± standard error) log CFU/mL (Figure 1A). The corresponding counts of this bacterium endospore after 3, 7, and 11 min of treatment at the above-mentioned intensity/temperature were reduced (*p* < 0.05) to 3.43 ± 0.22, 4.02 ± 0.35, and 2.57 ± 0.14 log CFU/mL, respectively (Figure 1A). The addition of 50K IU nisin was able to augment the decontamination efficacy of the treatment (Figure 1B). Before the treatment with 650 MPa at 50 °C and in the presence of 50K IU nisin, the counts of the *B. amyloliquefaciens* endospores were 6.92 ± 0.39 log CFU/mL (Figure 1B). The log reductions (*p* < 0.05) associated with 3, 7, and 11 min treatments were 3.06, 2.32, 4.07 logs, respectively (Figure 1B). The addition of lysozyme (224 mg/L) was as well efficacious to enhance the efficacy of the treatment. The endospore count of *B. amyloliquefaciens* before treatment with 650 MPa at 50 °C and in the presence of lysozyme (224 mg/L) was 6.84 ± 0.39 log CFU/mL (Figure 1C) and was reduced (*p* < 0.05) by 3.45, 2.84, and 4.60 logs after 3, 7, and 11 min of treatment (Figure 1C). It is noteworthy that currently in the private food industry, the vast majority of commercial treatments are designed to last three minutes [19,20,46]. Our study shows that extending this treatment to longer durations, if economically feasible, could have additional denomination benefits. Our study further illustrates that the application of nisin and lysozyme could augment the decontamination efficacy of a pressure-based treatment for the inactivation of bacterial endospores.

As discussed earlier, the current literature considers endospores of *B. amyloliquefaciens* TMW 2.479, which are used in this study, as one of the most-pressure resistant bacterial endospores. However, we observed that through synergism of mild heat (50 °C), bacteriocin and bactericidal compounds (nisin and lysozyme) and elevated hydrostatic pressure at 650 MPa, *B. amyloliquefaciens* endospores could be eliminated by >4.0 log CFU/mL (i.e., >99.99%). In contrast, we observed that *G. stearothermophilus* (ATCC^®^ 7953^TM^) was appreciably more resistant in response to the treatments. Endospore counts of *G. stearothermophilus* before treatments with 650 MPa (50 °C), with 650 MPa (50 °C) and 50K IU nisin, and with 650 MPa (50 °C) and 224 mg/L lysozyme were 5.05 ± 0.12, 6.03 ± 0.60, 5.12 ± 0.38 log CFU/mL, respectively (Figure 1). Neither of these three treatments tested for 3, 7, and 11 min was (*p* ≥ 0.05) capable of reducing the endospores of the microorganism. After 11 min of treatments at 650 MPa (50 °C), at 650 MPa (50 °C) with nisin, and 650 MPa (50 °C) with lysozyme, the counts of the endospore suspension were unchanged (*p* ≥ 0.05) and were 5.33 ± 0.16, 4.81 ± 1.10, 5.07 ± 0.14 log CFU/mL, respectively (Figure 1A–C). Considering the results of the current study, perhaps the endospores of *G. stearothermophilus* (ATCC^®^ 7953^TM^) could be considered one of the most pressure-resistant strains in future validation studies. This bacterium is currently considered as an indicator microorganism for gas and steam sterilization and also is traditionally associated with spoilage (causative agent of “flat sour”) of canned foods [30,31]. It is noteworthy that at a pressure intensity level of >600 MPa and temperatures >100 °C, modest reductions of *G. stearothermophilus* have been reported in the literature [47].

As discussed in Section 2.1., *B. atrophaeus* is considered an indicator microorganism for dry heat and steam sterilization treatments and had been only modestly studied in the high-pressure processing literature. This avirulent strain had also been proposed as a suitable surrogate for *B. anthracis*, the causative agent of anthrax, thus having importance also from a public health perspective [32]. Under the condition of our experiment conducted in carrot juice, we observed that endospore suspension of *B. atrophaeus* has comparable or more susceptibility to elevated hydrostatic pressure and nisin and lysozyme relative to *B. amyloliquefaciens* endospores (Figure 1A–C). Thus, if pressure-based pasteurization is validated against the endospores of *B. amyloliquefaciens*, the treatment will almost certainly eliminate the endospores of *B. atrophaeus* as well.

In the absence of any bacteriocin or bactericidal and at a pressure intensity level of 650 MPa (at 50 °C), treatments for 3, 7, and 11 min resulted in 3.78, 3.93, and 3.95 logs CFU/mL reductions (*p* < 0.05) in *B. atrophaeus* endospore suspension in carrot juice, respectively (Figure 1A). The same treatment in the presence of 50K IU nisin resulted in reduction (*p* < 0.05) of 4.29, 4.35, and 3.90 log CFU/mL, respectively (Figure 1B), and in the presence of 224 mg/L of lysozyme, it resulted in reductions (*p* < 0.05) of 2.92, 3.10, and 3.94 log CFU/mL, respectively (Figure 1C).

Overall results of the pressure treatments in carrot juice illustrate that resistance of the tested strains was in the order of *G. stearothermophilus*> *B. amyloliquefaciens*≥ *B. atrophaeus*. The addition of nisin and lysozyme provided further augmentation in the decontamination efficacy of treatments against *B. amyloliquefaciens* and *B. atrophaeus*. Pressure-based treatments with and without tested bacteriocin and bactericidal compounds were not efficacious for eliminating the endospores of *G. stearothermophilus*.

The result of the current study is in harmony with previous literature where treatments of up to 11 min were efficacious for the elimination of *B. amyloliquefaciens* endospores and 3 min treatments resulted in modest reductions of the bacterial endospore suspension in distilled water [27]. Studying pressure intensity levels of up to 700 MPa at temperatures ranging from 35 to 105 °C, other researchers also observed similar trends for pressure-based inactivation of *B. amyloliquefaciens* [48]. It is noteworthy that the current study and the vast majority of the units in commercial food processing utilize elevated hydrostatic pressure (i.e., water as the pressure transmission medium) while the vast majority of the discussed studies from the literature are derived from units with water–glycerol and/or mineral oil as a pressure transmission fluid. Thus, exercising caution is recommended for the interpretation and adoption of the studies from the literature that do not utilize hydrostatic pressure to ensure that validation studies have a high level of external validity.

### 3.2. Pressure-Based Reduction of Three Bacterial Endospores in Presence of Lactic Acid and Citricidal

The second set of experiments discussed in this section differs from previous trials as the bacterial endospores were used at a lower inoculation level, and sterilized distilled water (total dissolved solids <10 mg/L (ppm)) was used as the vehicle for the endospores. Additionally, 1% lactic acid and 1% Citricidal^TM^, a novel antimicrobial extracted from grapefruit seeds [49], were used in these experiments (Figure 2A–C).

Before the treatments, the counts of *B. amyloliquefaciens* endospores were 4.57 ± 0.22 log CFU/mL (Figure 2A). These counts were reduced (*p* < 0.05) by 2.02, 2.59, and 3.60 log CFU/mL after 3, 7, and 11 min of treatment at 650 MPa (50 °C), respectively (Figure 2A). Similar to the experiment conducted in carrot juice, the endospore counts of *B. atrophaeus* inoculated in distilled water exhibited comparable pressure-sensitivity trends relative to *B. amyloliquefaciens*. The endospore counts of *B. atrophaeus* before treatment (control) and after 3, 7, and 11 min of treatment at 650 MPa (50 °C) were 3.70 ± 0.47, 2.31 ± 0.23, 1.94 ± 0.16, and 2.17 ± 0.27, respectively (Figure 2A). Unlike these two strains, the counts of *G. stearothermophilus* endospore suspension remained unchanged (*p* ≥ 0.05) after the treatment at 650 MPa and 50 °C (Figure 2A). These results are in harmony with the experiment conducted in carrot juice where *G. stearothermophilus* exhibited a very high level of resistance to pressure treatments while the *B. amyloliquefaciens* and *B. atrophaeus* exhibited comparable sensitivity to elevated hydrostatic pressure (Figure 2A). The addition of 1% (*v*/*v*) lactic acid, however, to a great extent augmented the decontamination efficacy of the treatment against all three endospore suspensions, including *G. stearothermophilus* (Figure 2B). The control counts of treated samples at 650 MPa (50 °C) with 1% lactic acid were 4.67 ± 0.19, 4.57 ± 0.22, and 4.43 ± 0.24 for *B. amyloliquefaciens*, *G. stearothermophilus*, and *B. atrophaeus* endospore counts, respectively (Figure 2B). After a 3 min treatment, for the above order of microorganisms, these counts were reduced (*p* < 0.05) by 1.99, 2.02, and 2.06 log CFU/mL, respectively (Figure 2B). Longer treatment times provided additional decontamination benefits, whereas treatments for 7 min resulted in 2.44, 2.59, and 2.72 log reductions (*p* < 0.05), and treatments for 11 min lead to reductions (*p* < 0.05) of 2.95, 3.60, and 2.70 logs CFU/mL, for *B. amyloliquefaciens*, *G. stearothermophilus*, and *B. atrophaeus* endospore counts, respectively (Figure 2B).

These results indicate that lactic acid could, to great extent, augment the decontamination efficacy of the treatment against the endospore of these three indicator microorganisms and most importantly against *G. stearothermophilus* that exhibited very high resistance to elevated hydrostatic pressure treatments (Figure 2A,B). Lactic acid is a very common antimicrobial in food commerce; it has been tested against an array of pathogenic microorganisms and is the dominant antimicrobial of choice in North America’s meat industry [17]. This antimicrobial is also part of the recommendation of regulatory agencies, such as the World Health Organization, to improve the microbiological properties of food products [50]. It is noteworthy that lactic acid in many industries, including North America’s meat industry, has been utilized as a processing aid, i.e., applied during the process and then removed/rinsed by subsequent treatment. The current study utilized lactic acid as an antimicrobial agent that could be part of the formulation. Although this antimicrobial has the regulatory status of “generally recognized as safe,” [51] understandably it could nonetheless alter the physicochemical and organoleptic properties of a product during the shelf-life. Thus, further shelf-life and sensory testing are recommended before incorporating the utilization of lactic acid as an antimicrobial for augmenting the pressure-based treatment of microbial endospores.

Citricidal^TM^, a novel antimicrobial compound extracted from grapefruit seeds [49], however, did not provide any augmentation in decontamination efficacy of the treatment at 650 MPa (50 °C). The endospore counts of *B. amyloliquefaciens* before treatment (0 min i.e., control) and after 3, 7, and 11 min of treatment at 650 MPa (50 °C) in the presence of 1% Citricidal^TM^ were similar to those counts obtained by the treatment without the antimicrobial and were 4.73 ± 0.17, 2.71 ± 0.30, 2.13 ± 0.12, and 2.37 ± 0.20 log CFU/mL, respectively (Figure 2C). Similarly, endospore suspension counts of *B. atrophaeus* were reduced by (*p* < 0.05) 1.56, 1.94, and 2.09 log CFU/mL after treatments with 650 MPa (50 °C) in the presence of 1% Citricidal^TM^ for 3, 7, and 11 min, respectively (Figure 2C). These reductions were comparable to the reductions obtained from treatments at 650 MPa (50 °C) without any antimicrobial (Figure 2A). Endospore counts of *G. stearothermophilus* were not affected (*p* ≥ 0.05) by the treatment with 650 MPa (50 °C) in the presence of 1% Citricidal^TM^. These counts were 4.89 ± 0.17, and 4.84 ± 1.13 log CFU/mL before the treatment and after an 11-min treatment, respectively (Figure 2C). Although the application of Citricidal^TM^ and lactic acid to augment the efficacy of pressure-based treatments of bacterial endospores are only modestly investigated in the past, the results of the current study are in harmony with existing literature. It had been demonstrated in the past that with holding times as long as 10 min and pressure intensity levels of 600 MPa, low pH combined with pressure could reduce up to >3 logs of *B. amyloliquefaciens* and *G. stearothermophilus* [31]. In the absence of added bacteriocin and/or bactericidal compounds, others have concluded that although elevated pressure could reduce bacterial endospores, the method alone is not sufficient for full inactivation of common bacterial endospores. They additionally observed that bacterial endospores are considerably more resistant than fungal spores [52].

### 3.3. Linear and Non-Linear Inactivation Indices for the Reduction of Bacterial Endospores

Results obtained from the calculation of linear and non-linear inactivation indices are in concordance with the bacterial endospore reduction counts (Table 1). The current study calculated the linear D-value as the reciprocal of the positive slope of the best-fitted model resulting from plotting of endospore counts (log CFU/mL) as affected by the treatments of up to 11 min. Thus, the D-value corresponds to the amount of time (in minutes) required for one log (i.e., 90%) reduction of the microbial population. It is noteworthy that the D-value was calculated after enumerating the cells on a non-selective medium (tryptic soy agar) supplemented with 0.6% yeast extract to enhance recovery of pressure, heat, and antimicrobial injured cells. Non-linear K_max_ values were additionally calculated as an expression of numbers of log cycles of reduction, and since the unit of this index is 1/min, larger K_max_ values correspond to a lower inactivation rate and vice versa.

For the experiments conducted in carrot juice, the D-value for treating *B. amyloliquefaciens* endospores at 650 MPa (50 °C) was 3.45 min. This value was reduced to 2.85 min when 224 mg/L lysozyme was added to the treatment (Table 1). Similarly, the D-value of 2.82 was calculated for the inactivation of *B. atrophaeus* endospores for treatments at 650 MPa (50 °C). The non-linear inactivation indices also exhibited similar trends for the inactivation of endospores of *B. amyloliquefaciens* and *B. atrophaeus* (Table 1).

As further detailed in Section 3.1 and Section 3.2, while the addition of antimicrobials provided decontamination benefits for the reduction of the endospores for short-term processes, there were no appreciable differences among these endospore reductions of 3, 7, and 11 min treatments. In other words, while extending treatment time and the use of antimicrobials were able to augment the decontamination efficacy of the treatment, the application of both simultaneously did not provide added endospore reduction benefit. This indicates that the addition of bacteriocin and/or bactericidal compounds could augment the decontamination efficacy of the short-term treatments but do not necessarily provided added decontamination benefit for treatments longer than 5 min. This was further confirmed comparing the D-values of *B. amyloliquefaciens* after pressure treatment, pressure treatment with nisin, and pressure treatment with lysozyme where all three treatments exhibited similar D-values after treatments of up to 11 min. Lactic acid, however, was able to reduce the bacterial endospore inactivation indices of *B. atrophaeus*. The D-value for *B. atrophaeus* endospores after pressure treatment was 7.88 and was reduced when the microorganism was treated in the presence of 1% lactic acid. D-value for *G. stearothermophilus* was substantially reduced in the presence of 1% lactic acid. Similar trends were also observed based on non-linear inactivation indices (Table 1).

It is noteworthy that discussions on mechanisms of action of elevated hydrostatic pressure synergized with bacteriocin and bactericidal compounds for the elimination of bacterial endospores are very modestly presented in the literature [53,54]. The use of bacteriocins such as nisin that are historically associated with increasing the cortex hydrolysis of endospores during germination and identifying other efficacious antimicrobials, such as those explored in this study, could ensure the microbiological safety and economic feasibility of pressure-treated commodities [55]. Acidic antimicrobials, such as lactic acid used in this study, could have additional effects on endospore cortex hydrolysis and in augmenting their sensitivity to elevated hydrostatic pressure [56]. To ensure further success of this emerging technology for improving shelf-stability and microbial safety of an array of products, it is imperative for future studies to closely identify mechanisms of action and pathways associated with inactivation of bacterial endospores under elevated hydrostatic pressure and in the presence of bacteriocin and bactericidal compounds.

## 4. Conclusions

Under the conditions of our experiments, we observed that a well-design pressure treatment, synergized with mild heat and bacteriocin and/or bactericidal compounds, could reduce up to >4 log CFU/mL (i.e., > 99.99%) of bacterial endospores. More specifically, the addition of nisin and lysozyme were able, to great extent, to augment the decontamination efficacy of pressure-based treatments against *B. amyloliquefaciens* and *B. atrophaeus*, while no added benefit for reducing endospores of *G. stearothermophilus* was exhibited. Thus, the overall results of these pressure treatments illustrated that the resistance of the tested strains was in the order of *G. stearothermophilus* > *B. amyloliquefaciens* ≥ *B. atrophaeus*.

The addition of lactic acid, however, was efficacious for augmenting the pressure-based reduction of bacterial endospores of the above-mentioned three microorganisms. Citricidal^TM^, although validated in the past against planktonic cells of bacterial pathogens, exhibited no benefits in the reduction of the endospores of any of the microorganisms. Additionally, we observe that initial endospore load could be a determining factor in the success of treatment; thus, validation studies could consider mildly vs. highly contaminated food vehicles with bacterial endospores to assure external validity of validation studies.

Although many studies in the literature typically consider *B. amyloliquefaciens* as one of the most pressure-resistant spore-forming microorganisms associated with food products, we observed that *G. stearothermophilus* strain we utilized produced considerably more resistant endospores relative to *B. amyloliquefaciens.* Thus, *G. stearothermophilus* could be utilized in future microbiological pressure-based hurdle validation studies as a highly pressure-resistant indicator microorganism. We additionally observed that *B. atrophaeus* endospores have comparable sensitivity to pressure, heat, and antimicrobials relative to *B. amyloliquefaciens* endospores; thus, these could be used interchangeably in future validation studies. Utilization of *B. atrophaeus* endospores in hurdle validation studies could have additional co-benefits as well, since this microorganism is considered as a reliable surrogate for an important pathogen of public health concern, *B. anthracis*, the causative agent of anthrax. Finally, the food industry currently relies on treatments typically lasting for 3 min for pressure-treated commodities. Our study illustrates that extending this treatment time could provide additional decontamination benefits for the elimination of microbial endospores. An alternative to extending the treatment time could be the utilization of bacteriocin and/or bactericidal compounds to augment the efficacy of a pressure-based treatment for improving the microbiological profile of a product and extending shelf-life while assuring economic feasibility of the manufacturing. The addition of bacteriocin and/or bactericidal compounds adds the co-benefit of providing residual protection during the shelf-life of the product as well.

## Figures and Tables

**Table 1:** Inactivation indices for *B. amyloliquefaciens*, *G. stearothermophilus*, and *B. atrophaeus*.

Pathogen	Treatement ^a^	Medium	D-Value ^b^	K_max_^c^	R^2^	Std Error
*B. amyloliquefaciens*	HPP	Carrot Juice	3.45	0.67	0.57	0.15
	HPP + Nisin	Carrot Juice	3.34	0.83	0.48	0.23
	HPP + Lysozyme	Carrot Juice	2.85	1.01	0.62	0.21
*G. stearothermophilus*	HPP	Carrot Juice	- ^d^	-	-	-
	HPP + Nisin	Carrot Juice	8.18	0.28	0.11	0.16
	HPP + Lysozyme	Carrot Juice	41.84	0.51	0.36	0.31
*B. atrophaeus*	HPP	Carrot Juice	2.82	0.98	0.62	0.21
	HPP + Nisin	Carrot Juice	3.61	0.56	0.26	0.40
	HPP + Lysozyme	Carrot Juice	3.06	0.93	0.50	0.29
*B. amyloliquefaciens*	HPP	Distilled Water	3.32	0.69	0.81	0.25
	HPP + Lactic Acid	Distilled Water	4.11	0.56	0.12	0.57
	HPP + Citricidal^TM^	Distilled Water	5.07	0.45	0.48	0.12
*G. stearothermophilus*	HPP	Distilled Water	131.58	0.02	<0.1	0.21
	HPP + Lactic Acid	Distilled Water	14.33	0.03	<0.1	0.20
	HPP + Citricidal^TM^	Distilled Water	24.33	0.04	<0.1	0.12
*B. atrophaeus*	HPP	Distilled Water	7.88	0.29	0.21	0.13
	HPP + Lactic Acid	Distilled Water	4.39	0.52	0.57	0.11
	HPP + Citricidal^TM^	Distilled Water	5.79	0.40	0.48	0.10

^a^ HPP= treated with elevated hydrostatic pressure of 650 MPa at 50 °C, without any antimicrobial. HPP + Nisin = treated with elevated hydrostatic pressure of 650 MPa at 50 °C with 50K IU (*w*/*v*) nisin. HPP + Lysozyme = treated with elevated hydrostatic pressure of 650 MPa at 50 °C with 224 mg/L (*w*/*v*) lysozyme. HPP + Lactic Acid = treated with elevated hydrostatic pressure of 650 MPa at 50 °C with 1% (*v*/*v*) lactic acid. HPP + Citricidal^TM^ = treated with elevated hydrostatic pressure of 650 MPa at 50 °C with 1% (*v*/*v*) Citricidal^TM^. ^b^ *D*-value (min) was calculated and determined as the reciprocal of the positive slope of the best-fitted model (goodness-of-fit indicator of R^2^ values, α = 0.05), resulting from plotting of endospore counts (log CFU/mL) as affected by treatments. The counts were recorded from non-selective medium, supplemented with 0.6% yeast extract for recovery of heat, pressure, and antimicrobial injured cells. ^c^ K_max_ values (1/min) are selected using the GInaFiT software. K_max_ values indicate the expressions of number of log cycles of reduction in 1/min unit. ^d^ No endospore reduction was observed; thus, the inactivation index could not be calculated.

## Data Availability

Additional information and project data could be requested from the public health microbiology laboratory by contacting our program. The program website is: https://publichealthmicrobiology.education/ accessed on 1 March 2021.
